# Comparative Genotypic Analysis of RAPD and RFLP Markers for Molecular Variation Detection of Methicillin-Resistant *Staphylococcus aureus* Clinical Isolates

**DOI:** 10.3390/medicina58091245

**Published:** 2022-09-08

**Authors:** Fathy M. Elkady, Abdulaziz A. Al-Askar, Ahmed Abdel Tawab, Mohammad M. Alkherkhisy, Amr A. Arishi, Amr H. Hashem

**Affiliations:** 1 Microbiology and Immunology Department, Faculty of Pharmacy (Boys), Al-Azhar University, Cairo 11884, Egypt; 2 Department of Botany and Microbiology, Faculty of Science, King Saud University, Riyadh 11451, Saudi Arabia; 3 Department of Microbiology and Immunology, Faculty of Medicine, Al-Azhar University, Cairo 11884, Egypt; 4 School of Molecular Sciences, The University of Western Australia, Perth, WA 6009, Australia; 5 Botany and Microbiology Department, Faculty of Science, Al-Azhar University, Cairo 11884, Egypt

**Keywords:** *S. aureus*, MRSA, *coa* gene polymorphism, RFLP, RAPD

## Abstract

*Background and Objectives:* Methicillin-resistant *Staphylococcus aureus* (MRSA) isolates are associated with various diseases ranged from mild superficial impairments to invasive infections. This study aimed to evaluate the ability of polymerase chain reaction (PCR) based methods namely, restriction fragment length polymorphism (RFLP) of the *coa* gene and random amplified polymorphic DNA (RAPD), to determine the genetic diversity of MRSA isolates. Materials and Methods: A total of 37 MRSA isolates were conventionally identified depending on their biochemical and microbiological culture characteristics. Genotypic confirmation was based on detection of the associated *mec*A gene. The genetic variation amongst MRSA isolates was evaluated following the *coa* gene-based RFLP and RAPD fingerprints. *Results:* Results illustrated that, the species specific *coa* gene was detected in all MRSA isolates. The irregular bands intensity, number, and molecular sizes of the PCR amplicons demonstrated the *coa* gene polymorphism. The incompatible *Alu*I digestion patterns of these amplicons classified the tested MRSA isolates into 20 RFLP patterns which confirm the *coa* gene polymorphism. Additionally, the PCR-based RAPD analysis showed variable bands number with size range of approximately 130 bp to 4 kbp, which indicated the genetic variation of the tested MRSA isolates as it created 36 variable RAPD banding profiles. *Conclusions:* *coa* gene *Alu*I enzymatic restriction sites, amongst the tested MRSA isolates, certify their genetic variation on the basis of the accurate but complicated and relatively expensive *coa* gene-based RFLP. Conversely, the results verified the excellent ability of the simple and cost-effective PCR-based RAPD analysis to discriminate between MRSA isolates without any preface data about the genome.

## 1. Introduction

*Staphylococcus aureus* (*S. aureus*), a considerable Gram-positive opportunistic bacterium, may affect human skin, heart, bone, ear, and respiratory tract. This pathogen is associated with various diseases ranging from mild superficial impairment to serious, invasive, and life-threatening infections [1]. Some *S. aureus* strains have acquired the *mec*A gene, coding for altered penicillin-binding protein, with the consequent worldwide emergence of methicillin-resistant *S. aureus* (MRSA) strains. This altered protein is characterized by a low affinity for binding to all β-lactams which diminishes its clinical usefulness in controlling hospital and community acquired MRSA infections [2]. The widespread occurrence of *S. aureus* infections is due to multiple virulence factors of the pathogen besides their abundance in the environment and in normal microbiota [3]. All *S. aureus* strains have a unique capability to manipulate the coagulation process. Induction of human blood clotting formation represents one of the essential virulent activities associated with their pathogenesis. This plasma clotting activity is directly caused by the production of coagulase enzymes that catalyse the fibrin generation via prothrombin activation. Moreover, the formation of fibrin may provide protection against phagocytosis [4]. Accordingly, coagulase enzymes created by all *S. aureus* strains are considered one of the fundamental phenotypic measures for the detection of their clinical infections [5]. These enzymes are found either extracellularly or membrane-bounded and encoded by the *coa* gene, one of the foremost species-specific virulent genes that are used for molecular confirmation of the diseases caused by *S. aureus* [6].

The Pulsed-Field Gel Electrophoresis (PFGE) is the gold standard technique for epidemiological investigation of microbial infections. Nevertheless, the comparatively high expense of performance and the requirement of specialized apparatus obstruct their use [7]. The inconsistent nature of the *coa* gene 3′ coding region is responsible for their highly polymorphic nature and subsequent variable amino acid sequence in coagulase protein. This region contains a chain of 81 base pair (bp) tandem repeats or short repetitive sequences coding for 27 amino acid repeated sequences. The number of these tandem repeats as well as the positions of restriction enzyme cutting sites varied amongst *S. aureus* isolates [8]. Consequently, this variation permits the discrimination and DNA-based genotyping of *S. aureus* isolates. The *coa* gene amplification, using DNA primer complementary to a definite sequence in its conserved region, produced dissimilar amplicon sizes in the tested isolates [9]. Additionally, analysis of the polymerase chain reaction-based restriction fragment length polymorphism (PCR-based RFLP) of *coa* gene amplicons can be used for *S. aureus* isolates genotyping. This technique depends on the dissimilarity in the sequence of the coding region at the 3′ end with subsequent variation in the sites of *Alu*I and *Hae*III restriction enzymes effect [10]. Moreover, the PCR-based random amplified polymorphic DNA (PCR-based RAPD) is a rapid, simple, and effortless technique at a low annealing temperature. It utilizes primers with arbitrary short oligonucleotides that able to randomly amplify various DNA targets. The created fragments vary in their numbers and sizes depending on the divergence in the length between the primer annealing sites that is unique for each bacterial isolate [11]. This method permits strain typing on the basis of a large part of their genomic DNA, independently on well-known data, and provides different banding profiles with subsequent better strain fingerprinting [12].

Herein, this search seeks to assess the capability of two PCR depending methods, the costly and laborious RFLP based on the *coa* gene and the inexpensive RAPD technique with little stringent conditions based on the whole genome, to detect the genomic variation and subsequent diversity of MRSA clinical isolates.

## 2. Materials and Methods

### 2.1. Isolation and Phenotypic Identification of S. aureus

Clinical specimens from hospitalized patients were instantly cultured on nutrient agar (Oxoid, Basingstoke, Hampshire, UK) and blood agar followed by incubation at 37 °C for 24 h. *Staphylococcus aureus* was phenotypically identified depending on their distinctive ability to grow on mannitol salt agar (Oxoid, Basingstoke, Hampshire, UK) with sugar fermentation. As well, their Gram stain appearance and biochemical behaviours, following standard microbiological procedures, including catalase, coagulase, and DNase production, were examined [13].

### 2.2. Phenotypic Recognition of MRSA

The MRSA clinical isolates were identified on the bases of the cefoxitin disc diffusion test following the Kirby–Bauer disk diffusion method using 0.5 McFarland standard bacterial suspension on Muller Hinton agar medium (Oxoid, UK). The inhibition zone diameter around the cefoxitin (30 µg) disc (Oxoid, UK) was determined after 18 h incubation at 35 °C [14].

### 2.3. Genotypic Confirmation of MRSA

Definite identification of MRSA isolates was dependent on the detection of their characteristic *mec*A gene [15]. Briefly, genomic DNA was extracted using GeneJET Genomic DNA Purification Kit according to the manufacturer’s instructions (Thermo Fisher Scientific Inc., Vilnius, Lithuania). The *mec*A gene was amplified, by PCR technique, using a Thermal cycler (Biometra UNO–Thermoblock, Germany) in presence of 1 µL from each particular 10 pmol primer (Table 1), 12.5 µL Cosmo PCR Red Master Mix (Willowfort, Birmingham, UK), 1 µL of 50 ng genomic DNA, and nuclease-free double distilled water to final reaction volume of 25 µL. The *mec*A gene amplification cycling program includes 1 cycle of initial denaturation at 94 °C for 4 min (min) followed by 35 cycles of denaturation at 94 °C for 1 min, annealing at 55 °C for 1 min, and elongation at 72 °C for 1 min, and lastly 1 cycle of final elongation at 72 °C for 10 min. The obtained PCR products were loaded into 1.5% agarose gel containing 5 µL ethidium bromide (0.05 mg/mL) followed by electrophoresis at 100 V in Tris-acetate EDTA buffer (Sigma Aldrich, Hamburg, Germany). The UV transilluminator (HERMLE Labortechnik GmbH, Wehingen, Germany) was used to observe the *mec*A gene-specific bands and determine their molecular size with the aid of a 100 bp DNA ladder (Geneaid Biotech Lt., New Taipei City, Taiwan) as a marker [16].

### 2.4. Coagulase (Coa) Gene Molecular Typing of MRSA Isolates

The coding region of the *coa* gene 3′ end was amplified by PCR using 1 µL from each 10 pmol primer (Table 1), 10 µL Cosmo PCR Red Master Mix (Willowfort, UK), 1 µL of 50 ng genomic DNA, and nuclease-free double distilled water to final reaction volume of 20 µL. The PCR cycling profile includes 1 cycle of initial denaturation at 95 °C for 2 min, 35 cycles of denaturation at 95 °C for 30 s (s), annealing at 55.5 °C for 30 s, and extension at 72 °C for 50 s, followed by 1 cycle of final extension at 72 °C for 5 min. *Staphylococcus aureus* ATCC 6538 was used as a positive control and PCR amplicons were detected with the aid of a UV transilluminator after 1.5% agarose gel electrophoresis in presence of a 50 bp DNA marker [6]. The PCR amplification products of *coa* gene were analysed after treatment with *Alu*I restriction enzyme (BioLabs, Inc., New England, MA, USA) following the manufacturer’s information. Briefly, 50 µL reaction mixture consists of 10 µL from each PCR product, 34 µL nuclease-free distilled water, and 6 µL of 10 U *Alu*I (1 µL of the enzyme in 5 µL rCutSmart buffer) was prepared and incubated for 15 min at 37 °C followed by enzyme inactivation by heating at 80 °C for 20 min. The RFLP pattern for each isolate was separated by electrophoresis using agarose gel 1.5% and matched with a 50 bp DNA marker [18].

### 2.5. PCR-Based RAPD Fingerprinting of MRSA Isolates

The random DNA fragments amplification reactions were achieved in a total reaction volume of 20 µL containing 10 µL Cosmo PCR Red Master Mix (Willowfort, UK), 1 µL of 50 ng genomic DNA, 2 µL of 25 pmol primer that targets the whole genome (Table 1), and 7 µL of nuclease-free double distilled water. The PCR amplification program involves the application of 1 cycle of initial denaturation at 94 °C for 5 min with subsequent 40 cycles of denaturation at 94 °C for 1 min, annealing at 40 °C for 1 min, and extension at 72 °C for 3 min, and to finish 1 cycle of final extension at 72 °C for 7 min. The RAPD pattern for each isolate was separated by electrophoresis using 1% agarose gel and visualized by a transilluminator and compared with 1 kbp plus DNA ladder (Enzynomics, Daejeon, South Korea) as a marker [17].

### 2.6. Phylogenetic Analysis

The variable bands number and size of the *coa* gene PCR-based RFLP and PCR-RAPD patterns of the tested MRSA isolates were separately analysed. Dice Similarity Coefficient and Unweighted Pair Group Method with Arithmetic Average (UPGMA) were used to construct the phylogenetic trees (dendrograms). The presence or absence of a specific band was coded by binary numbers of 1 (for presence) or 0 (for absence). Isolates with about 70% similarity in the dendrogram were collected in groups identified as clusters.

### 2.7. Statistical Analysis

The discriminatory power of each typing method was evaluated by calculating the Simpson’s index of diversity (discriminatory index (D)) according to the following equation’:

$$D=1-\frac{1}{N\left(N-1\right)}.\overset{s}{\underset{j=1}{\sum }}nj\;\left(nj-1\right)\;$$

where *N* is the total number of isolates in the sample population, *S* is the total number of the obtained types, and *nj* is the isolates number belonging to the most frequent type [19].

## 3. Results

### 3.1. Identification of MRSA

**Figure 1:**
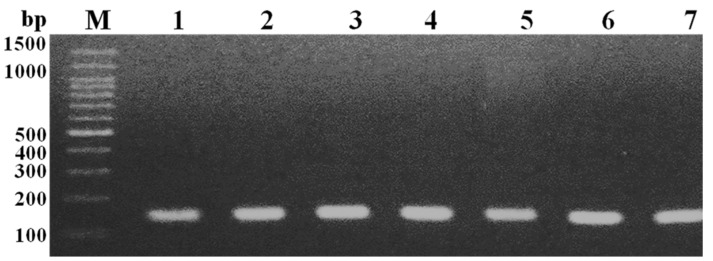
Agarose gel electrophoresis of *mec*A gene PCR amplicons at about 147 bp. Lanes, M; 100 bp DNA ladder and 1–7; showed the representative some positive results of *mec*A gene specific bands in phenotypic MRSA isolates 1–7, respectively.

A total of 37 Gram-positive cocci with a typical bunch of grapes-like appearance, a β-haemolytic character on blood agar, golden-yellow colonies on nutrient agar, and mannitol fermenting activity were assumed to be *S. aureus*. Additionally, the positive behaviours for catalase, coagulase, and DNase tests were biochemically confirmed their identification. Furthermore, these isolates were phenotypically emphasized as MRSA depending on their growth inhibition around cefoxitin disc (30 µg) at a diameter ≤ 21 mm. Genotypically, detection of the expected *mec*A gene-specific band, of approximately 147 bp (Figure 1), in all the 37 tested *S. aureus* isolates indicated their confirmed identification as MRSA.

### 3.2. PCR-Based RFLP of Coa Gene

**Figure 2:**
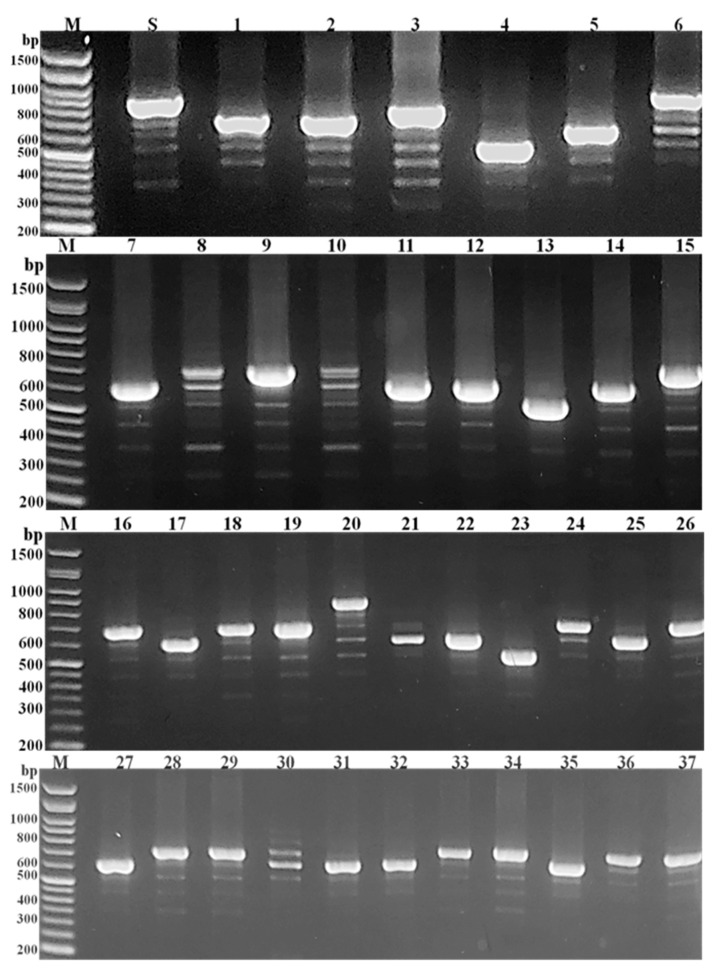
Agarose gel (1.5%) electrophoresis of *coa* gene PCR products. Lanes, M; 50 bp DNA ladder, S; *S. aureus* ATCC 6538, and 1 to 37; showed the *coa* gene variable bands size, number, and intensity in the tested MRSA isolates number 1–37, respectively.

**Figure 3:**
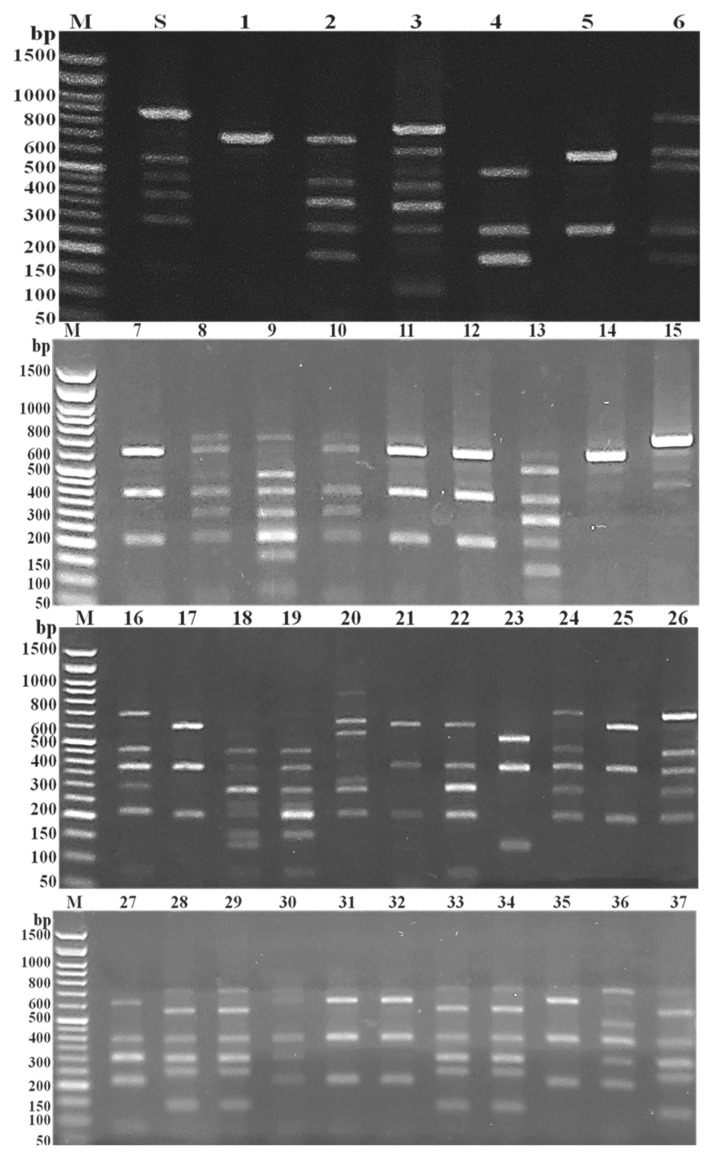
Agarose gel (1.5%) electrophoresis profiles of *coa* gene based *AluI* enzymatic digestion. Lanes, M; 50 bp DNA ladder, S; *S. aureus* ATCC 6538, and 1 to 37; showed the miscellaneous RFLP patterns of *coa* gene amongst the tested MRSA isolates number 1–37, respectively.

All the tested MRSA isolates were *coa* gene-positive with uneven PCR amplicon sizes. The *coa* gene polymorphism was demonstrated by the detection of their inconsistent band’s number (1 to 6 bands). Moreover, these bands showed variable intensity and molecular sizes of approximately 81 bp multiplied in the range of 243 to 972 bp (Figure 2). As well, the agarose gel electrophoresis of *Alu*I enzymatic digestion of the *coa* gene amplicons showed 3 to 7 DNA fragments. The size of each fragment was approximately equal to one of the 81 bp multiplied and in the range of 81 to 891 bp (Figure 3).

The incompatible digestion patterns confirm the *coa* gene polymorphism among the tested MRSA isolates and resulted in 20 diverse fragmentation classes nominated as genotype F_1_–F_20_. Amongst these genotypes, the majority of the tested isolates (9/37; 24.3%) were mostly related to genotype F_13_ (isolates number 7, 11, 12, 17, 21, 25, 31, 32, and 35) with three DNA fragments size of 648, 405, and 243 bp. Genotype F_7_ was contained 6/37 isolates (16.2%), isolates number 9, 28, 29, 33, 34, and 37, with six fragmentation sizes of 729, 567, 405, 324, 243, and 162 bp. Additionally, 3/37 isolates (8.1%) were included in genotype F_11_ (isolates number 10, 27, and 30) with four DNA fragments sizes of 648, 405, 324, and 243 bp. As well, genotype F_19_ included isolates number 24, 26, and 36 (3/37 isolates: 8.1%) with five fragmentation sizes of 648, 405, 324, 243, and 162 bp. On the other hand, the remaining 16 isolates revealed high degree of heterogeneity as each isolate represented a separate genotype with variable restriction patterns. Additionally, isolate number 1 showed an indigestible *coa* gene amplicon (genotype F_3_).

**Figure 4:**
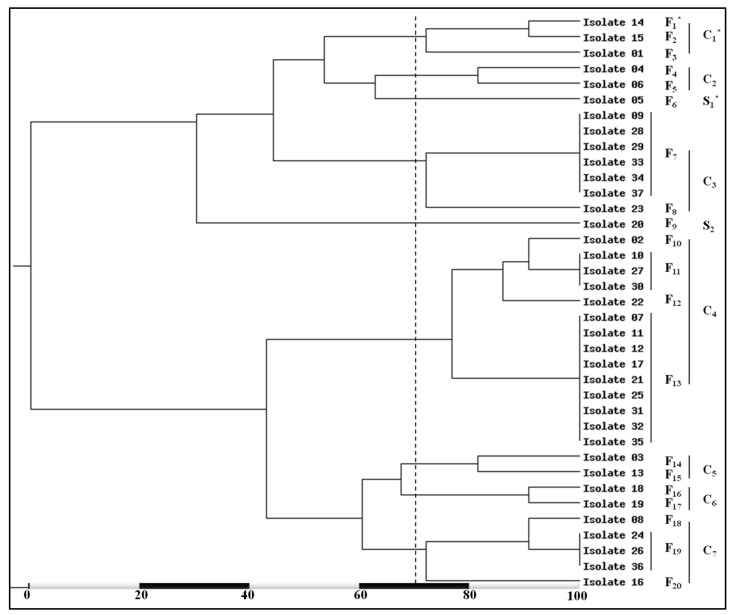
Dendrogram clarifying the variation amongst the tested MRSA isolates on the bases of *Alu*I enzymatic digestion of *coa* gene PCR amplicons. * F_1_–F_20_; the RFLP genotypes, C_1_–C_7_; the obtained clustered, and S_1_ and S_2_; the established singletons, based on approximately 70% similarity (dotted line).

The generated dendrogram, depending on 100% similarity, and its clusters showed variable similarity percentages (Figure 4). Based on approximately 70% similarity, the 20 RFLP genotypes were grouped into 7 RFLP clusters (C_1_–C_7_) and 2 singletons (S_1_ and S_2_). The highest percentage (37.8%; 14/37) of isolates were included in C_4_ (the most prevalent cluster) while, C_2_ (the lowest prevalent cluster) comprised only two isolate (5.4%).

### 3.3. RAPD Fingerprinting of MRSA Isolates

**Figure 5:**
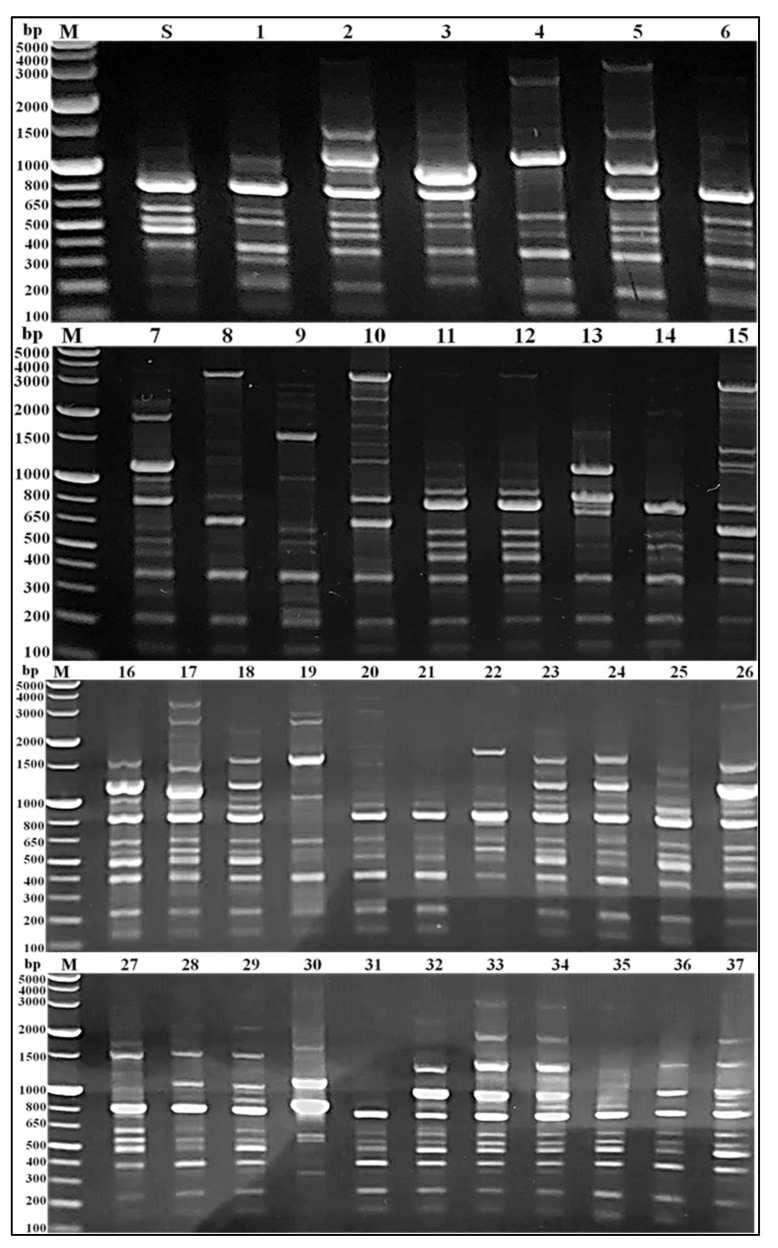
Agarose gel (1%) electrophoresis patterns of PCR-based RAPD. Lanes, M; 1 kbp plus DNA ladder, S; *S. aureus* ATCC 6538, and 1 to 37; showed the diverse RAPD patterns of the tested MRSA isolates number 1–37, respectively.

The genetic variation of the tested MRSA isolates was decided by PCR-based RAPD (Figure 5). The results showed several bands (6–18), with a molecular size range of approximately 130 bp to 4 kbp. As well, some conserved bands e.g., 220 bp and 400 bp were mostly detected in the tested isolates. The 220 bp bands were detected in all isolates except isolates number 22 and 30. While, bands with the molecular size of 400 bp were detected in all isolates except isolate number 30.

Importantly, nine unique bands of approximately 4000, 3500, 3100, 2700, 2600, 2100, 1700, 750, and 580 bp molecular size were detected isolates number 20, 17, 34, 19, 9, 14, 10, 13, and 30, respectively. Similarly, each of 10 distinctive bands with the molecular size of approximately 3600, 2800, 2300, 1900, 1850, 1750, 920, 600, 450, and 230 bp were observed in only two of the tested isolates; numbers (5 and 25), (15 and 16), (7 and 9), (7 and 33), (22 and 34), (15 and 27), (33 and 34), (10 and 30), (8 and 10), and (9 and 20), respectively.

The unpredictable appearance of the conserved and variable bands as well as, the changeable band’s numbers and sizes creates 36 variable RAPD banding profiles, voted genotypes B_1_–B_36_. The MRSA isolates number 11 and 12 represented the same RAPD banding profiles (B_11_) while, other tested MRSA isolates revealed a high level of variability as each isolate was represented only one RAPD profiles.

**Figure 6:**
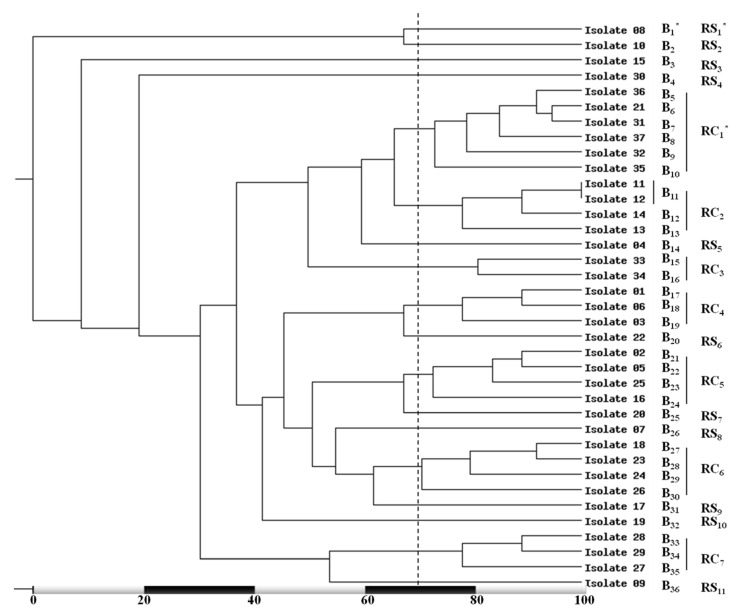
Dendrogram demonstrates the high level of genetic variation between the tested MRSA isolates on the bases of PCR-based RAPD banding patterns analysis. * B_1_–B_36_; the RAPD genotypes, RC_1_–RC_7_; the created clustered, and S_1_ and S_11_; the recognized singletons, based on approximately 70% similarity (dotted line).

Based on about 70% similarity, the phylogenetic analysis of the obtained 36 RAPD-PCR fingerprints revealed seven variable RAPD clusters (RC_1_–RC_7_) and 11 RAPD singletons (RS_1_–RS_11_). The results recorded RC_1_ as the most ubiquitous cluster which included six (16.2%) isolates while RC_3_ included only two isolates (5.4%) and represent the less common cluster (Figure 6).

### 3.4. Discrimination Index

The discriminatory power for each genotyping method was evaluated by calculation of their numerical index of discrimination. The Simpsons index of diversity showed the highest capability of RAPD-PCR, with a D value of more than 0.97, for isolates discrimination. On the other hand, the *coa* gene-based RFLP revealed lower discrimination ability, with a D value of 0.35.

## 4. Discussion

Methicillin-resistant *S. aureus* isolates represent one of the most considerable infectious agents with an increased tendency to resist a wide range of currently available antimicrobial agents [20]. Therefore, this study was performed to detect the MRSA isolates and to assess the capability of the *coa* gene-based RFLP and whole genome-based RAPD analysis for detection of their genetic variation. In the current study, thirty-seven *S. aureus* isolates were identified following conventional phenotypic microscopical, cultural, and biochemical characteristics. In a related study, *S. aureus* was identified based on their capability on mannitol fermentation, golden yellow endopigment production, β-haemolytic activity, and coagulase positive test [21]. The overspread and increased incidence of MRSA infections necessitate the urgent need for their rapid identification. In the present study, phenotypic cefoxitin resistance with subsequent *mec*A gene molecular finding was considered as a marker for MRSA isolates confirmation. A similar study carried out by Bokharaei, et al. [22] described the cefoxitin disc test and PCR amplification of the *mec*A gene as an effective tool for MRSA strains recognition.

Molecular detection of the *coa* gene, which presents in all phenotypically coagulase-positive *S. aureus* isolates, is an important genotypic measure for their identification. Additionally, recognition of this gene is an easy, precise, and discriminatory method for *S. aureus* isolates genotyping [23]. In this study, the *coa* gene was detected in all the tested MRSA isolates and its polymorphic nature was initially predicted. The PCR amplicons of the *coa* gene were inconsistent with variable bands number (1 to 6 bands), size (243 to 972 bp), and intensity. In a related study carried out in Egypt, up to eight bands of *coa* gene amplicons were recorded among the tested MRSA isolates with a band size range of 80 to 810 bp [24]. These results were in accordance with Al-Ruwaili [25] study who differentiate 11 *coa* gene band classes in the range of 500 to 1000 bp. The tested MRSA isolates showed unequal *coa* gene bands number (1 to 4 bands) with incompatible intensity. Insertion or deletion mutations, at the 3′ end, might be responsible for these *coa* gene polymorphism with subsequent change in the PCR amplification products size. Additionally, the coexistence of numerous *coa* gene allelic form allows a single MRSA isolate to produce several gene variants which explained the feasible formation of a number of bands by the same isolate [10].

Molecular typing methods depending on specific gene amplification with subsequent enzymatic digestion are used for MRSA isolates characterization. These genotyping methods exhibit valuable discrimination ability and are supportive in the determination of the variability amongst MRSA isolates on genetic bases [26]. In the current study, *coa* gene-based RFLP, using *Alu*I restriction enzyme, of the 37 tested MRSA isolates, yielded 20 banding patterns. These patterns exhibited 3 to 7 DNA fragments of approximately one of the 81 bp multiplied in the size range of 81 to 891 bp. Additionally, one isolate showed *coa* gene amplicon resistant to *Aul*I cutting. These results were relatively following a comparative study carried out by Al-Ajealy, et al. [27], they recorded seven *Aul*I restriction patterns of *coa* gene amplicons with 1 to 3 DNA fragments in a size range of 80 to 700 bp. Additionally, their results also recorded *Aul*I enzyme undigested *coa* amplicons in 20% of their tested isolates. Similarly, in the study carried out by Chadi Dendani, et al. [28], the PCR-based RFLP of *coa* gene showed nine different *Alu*I restriction patterns with approximately band sizes of 90 to 550 bp. The *S. aureus* isolates with faint *coa* amplification product yielded no detectable bands after *Alu*I enzymatic digestion. The obtained results disagreed with Ibrahim, et al. [29] who observed just three PCR-based RFLP patterns showed 3 to 4 DNA fragments with a size range of 80 to 500 bp. The existence of the *Alu*I target site in the *coa* gene sequence may reflect the ability of this enzyme to digest the *coa* gene amplicons in the susceptible isolates. Additionally, the altered *coa* gene sequence amongst diverse isolates provide dissimilar restriction locations [30]. Conversely, a point mutation at the 3′ region of *coa* gene and subsequent lack of *Alu*I enzyme-specific restriction sites may reflect the incapability of the *Alu*I enzyme to digest the *coa* gene PCR amplicon of the digestion resistant isolates [31].

Recently, epidemiological investigations of bacterial outbreaks on the basis of the whole genome may provide an incomparable resolution [26]. In the present study, 36 inconsistent RAPD banding patterns were observed amongst the 37 tested MRSA isolates with elevated variability levels. As well, these banding patterns were divided into seven RAPD clusters and 11 RAPD singletons. The study conducted by Mobasherizadeh, et al. [32] described the RAPD-PCR as a perfect, fast, simple, and low-cost genotyping technique depending on a small amount of total DNA. Their study classified 25 MRSA isolates into five genotypes based on RAPD-PCR. The tested isolates were divided into two clusters (groups each with more than three members), a small group containing three members, and single isolates each with distinctive RAPD-PCR patterns. In a related study, all the tested MRSA isolates were generating an abundance band, in the range of 400 to 1000 bp, by RAPD-PCR which subsequently divided the tested isolates into five different clusters [33]. The different frequencies and locations of the target sequences within the chromosomal DNA reflect the variation in DNA fragments number and size in different RAPD-PCR patterns with subsequent genetic variation depending on the sequence of the arbitrarily short primer used [34].

In the present study, the RAPD-PCR numerical index of discrimination of more than 0.97 illustrates their highest ability for isolates differentiation. Similarly, the study carried out by Ye, et al. [35] demonstrated the high capacity of RAPD-PCR for differentiation between *S. aureus* isolates with discriminatory index of about 0.95. The random reliance of PCR-based RAPD method on the whole bacterial genome might explain the excellent ability of such technique for isolates characterization as well as its usefulness as a molecular genotyping tool in the epidemiological investigation.

## 5. Conclusions

Bacterial genotyping represents an essential part of infection control measures. These methods help in outbreaks investigation and clarification of the infection source which might be another patient, staff members, apparatus, food, or the environment. The incompatible *coa* gene digestion patterns, among the tested MRSA isolates, validate their genetic inconsistency and illustrate the confirmatory discrimination ability based on the precise but relatively expensive and multi-step *coa* gene-RFLP typing method. In contrast, the present study verified the ability of the simple and inexpensive RAPD-PCR analysis to discriminate between MRSA isolates based on the whole bacterial genome without any preface information. As well, this technique provides an improved insight on MRSA isolates typing with consequent useful epidemiological investigations and superior patient concern.

## Figures and Tables

**Table 1:** Nucleotide sequences of the primers used in this study.

Target Sequence	Primer Sequence (3′ 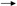 5′)	Amplicon Size (bp)	Reference
*mec*A gene	GTGAAGATATACCAAGTGATT ATGCGCTATAGATTGAAAGGAT	147	[15]
*coa* gene	ATAGAGATGCTGGTACAGG GCTTCCGATTGTTCGATGC	Variable	[6]
Whole-genome	GAGGGTGGCGGTTCT	Variable	[17]

## Data Availability

Not applicable.
